# EFFECT OF REPETITIVE PERIPHERAL MAGNETIC STIMULATION ON POSTOPERATIVE PAIN CONTROL IN FOOT AND ANKLE SURGERY

**DOI:** 10.1590/1413-785220263405e301789

**Published:** 2026-08-10

**Authors:** Eli Ávila Souza, Laura Pereira Generoso, Gabrielly Santos Pereira, Marcelo Lourenço da Silva, Alberto Cliquet

**Affiliations:** 1 Universidade Federal de Alfenas, Faculdade de Medicina, Departamento de Ortopedia e Traumatologia, Alfenas, MG, Brasil.; 2 Universidade Federal de Alfenas, Departamento de Fisioterapia, MG, Brasil.; 3 Universidade Estadual de Campinas (Unicamp), Faculdade de Ciências Médicas, Departamento de Ortopedia e Traumatologia, Campinas, SP, Brasil.

**Keywords:** Surgery, Pain, Magnetic Field Therapy, Foot, Ankle, Cirurgia, Dor, Magnetoterapia, Pé, Tornozelo

## Abstract

**Objective:**

This study aimed to investigate the effects of Repetitive Peripheral Magnetic Stimulation (rPMS) treatment on postoperative pain control after foot and ankle surgeries.

**Methods:**

A controlled, randomized clinical trial conducted at a tertiary hospital. 67 patients who underwent foot and ankle surgeries were randomized into two groups: a control group (CG) – placebo stimulation; and an experimental group (EG) – rPMS for 10 minutes using an intermittent stimulation protocol (10 cycles/5 seconds; 100 Hz; 55 seconds of rest). Stimulation was received for 5 consecutive days in the immediate postoperative period. Neuropathic Pain Score 4 (DN4), Visual Analog Scale (VAS), Brief Pain Inventory (BPI), and American Orthopedic Foot and Ankle Score (AOFAS) were administered on days D0, D5, D14, D30, and D90. Postoperative opioid consumption was compared between the groups.

**Results:**

The most prevalent surgeries were in the forefoot (n=25). On DN4, the predominant pain was nociceptive (3.40 ± 1.54). Regarding pain, the VAS, BPI, and AOFAS scores demonstrated superior improvement in pain perception in the EG compared to the CG, with statistical significance. rPMS reduced postoperative opioid consumption.

**Conclusion:**

rPMS contributes to the control and reduction of pain perception and opioid consumption in the postoperative period of foot and ankle surgeries. ***Level of Evidence I; Randomized Clinical Trial.***

## RESUMO

**Objetivo:**

Este estudo teve como objetivo investigar os efeitos do tratamento com Estimulação Magnética Periférica Repetitiva (rMPS) no controle da dor pós-operatória em cirurgias de pé e tornozelo.

**Métodos:**

Ensaio clínico controlado e randomizado, realizado em um hospital terciário. Sessenta e sete pacientes submetidos a cirurgias de pé e tornozelo foram randomizados em dois grupos: grupo controle (GC) – estimulação placebo; e grupo experimental (GE) – EMpr por 10 minutos com protocolo de estimulação intermitente (10 ciclos/5 segundos; 100 Hz; 55 segundos de repouso). A estimulação foi aplicada por 5 dias consecutivos no pós-operatório imediato. O Neuropathic Pain Score 4 (DN4), a Escala Visual Analógica (EVA), o Brief Pain Inventory (BPI) e o American Orthopedic Foot and Ankle Score (AOFAS) foram administrados nos dias D0, D5, D14, D30 e D90. O consumo de opioides no pós-operatório foi comparado entre os grupos.

**Resultados:**

As cirurgias mais prevalentes foram no antepé (n=25). No DN4, a dor predominante foi nociceptiva (3,40; ±1,54). Em relação à dor, os escores EVA, BPI e AOFAS demonstraram melhora superior na percepção dolorosa no GE em comparação ao GC, com significância estatística. A rMPS reduziu o consumo de opioides no pós-operatório.

**Conclusão:**

A rMPS contribui para o controle e a redução da percepção dolorosa e do consumo de opioides no pós-operatório de cirurgias de pé e tornozelo. ***Nível de Evidência I; Ensaio Clínico Randomizado.***

## INTRODUCTION

Foot and ankle surgical pain can greatly impact recovery^1,2^. Postoperative pain levels depend on surgery type, patient factors, and pain management effectiveness^3^. Inadequate postoperative pain control is associated with longer postoperative recovery room stays and longer hospital stays^4,5^, impacting mobility, rehabilitation progress, and quality of life^3^. Despite advances in multimodal analgesia, moderate to severe postoperative pain affects up to 80% of patients^6^.

In the management of postoperative pain after foot and ankle surgeries, measures such as peripheral nerve blocks, wound infiltrations, joint infiltration with glucocorticoids, and oral medications (paracetamol, nonsteroidal anti-inflammatory drugs, selective cyclooxygenase-2 inhibitors, and opioids) are considered^5,7,8^.

Repetitive Peripheral Magnetic Stimulation (rPMS) is an innovative therapeutic modality for pain control through peripheral neuromodulation. It is a non-invasive treatment method developed for therapeutic neuromodulation in movement disorders.

In rPMS, an electromagnetic coil generates a strong magnetic field that induces electrical currents to influence nearby neurons. The effects vary based on coil type and orientation, application site, stimulation frequency, and session number^9^. rPMS therapy provides pain-free stimulation to deep muscle tissues^10^. It can activate peripheral nerves and muscle spindles even through layers of skin, fat, bone, clothing, or other materials. The technique is painless, non-invasive, and simple to use^11,12^, making it a safe way to target deeper muscles without discomfort. These advantages make the technique suitable for safely stimulating postoperative areas ^12,13^.

The aim of the study is to investigate the effects of rPMS treatment on postoperative pain control after foot and ankle surgeries.

## MATERIAL AND METHODS

### Study design and setting

Controlled, randomized clinical trial with sixty-seven individuals treated in a tertiary hospital in Brazil.

### Participants

Inclusion criteria: Individuals in the immediate postoperative period of foot and ankle surgeries; individuals 18 years of age or older.

Exclusion criteria: Individuals under 18 years of age; those not experiencing postoperative pain; those with malignant neoplasms or infections; pregnancy; vascular, neurological, or skin diseases affecting the lower limbs; and individuals with cognitive impairment were excluded from the study.

### Randomization and sample size

The sample size calculation was based on a pilot study of 24 individuals. Statistical analyses were performed using R software, considering α = 5% and β = 80%. The minimal sample size was determined for each variable (VAS, IBD, and AOFAS) according to their standard deviations and desired mean differences. The VAS variable required the largest sample, resulting in a minimum of 33 participants per group (n = 66 total) to ensure adequate statistical power.

Participants were assigned to either the control group (CG) or experimental group (EG) using simple randomization via Research Randomizer (version 4.0)^14^. Random numbers ensured balanced allocation, and a researcher uninvolved in data collection or analysis conducted the process for sequence concealment and reduced selection bias.

The following variables were considered to characterize the sample: sex; age; type of surgery (bone vs. soft tissue); area (ankle, hindfoot, midfoot, forefoot); smoking status; functionality; surgical history; duration of surgery; anesthesia; tourniquet use.

### Clinical setting and procedures

All patients included in the study received a standardized analgesic prescription for the postoperative period to ensure homogeneity in pain management between groups: 1g dipyrone every 6 hours, plus 50 mg tramadol every 8 hours if severe pain occurred. Patients were informed of the regimen and instructed to track tramadol use during the first two weeks after surgery. These records were submitted to the lead researcher for opioid consumption analysis.

**Figure 1:**
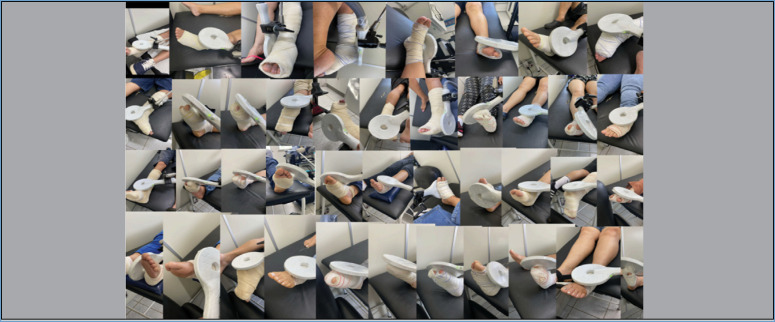
EMPr application area.

Source: Study author.

Repeated Peripheral Magnetic Stimulation (rPMS): Study participants were randomized into two groups: EG (n=34) and CG (n=33). Both groups used the rPMS device. Peripheral electromagnetic neuromodulation sessions were performed in an outpatient setting, with the patient comfortably positioned supine on a stretcher, maintaining the operated limb in a neutral position. The coil used was a figure-of-eight coil, coupled to the NEURO-MSX stimulator (Neurosoft®, UK), and fixed over the anatomical area corresponding to the surgical topography. The coil was positioned so that its surface was parallel to the operated region, 1cm away. For the EG, 5 neuromodulation sessions were performed, with a frequency set at once daily, for 5 consecutive days, starting on the fifth postoperative day. An intermittent stimulation protocol consisted of 10 cycles of 5 seconds of stimulation at a frequency of 100 Hz, followed by 55 seconds of rest, with a minimum intensity of 20% up to maximum intensity. In the CG, the coil was applied transversely to the surgical wound, but there was no stimulation (Figure 1). Both groups heard the same clicking sound during sessions, with the coil placed in contact for a similar sensation. Experienced physical therapists administered rMPS to both groups under the main surgeon's supervision.

### Assessment instruments

Neuropathic Pain 4 (DN4)^15,16^: Used to classify postoperative pain after foot and ankle surgery (nociceptive or neuropathic) and correlate it with the different anatomical areas of the foot and ankle (ankle, hindfoot, midfoot, and forefoot).

Visual Analog Scale (VAS)^17^, Brief Pain Inventory (BPI)^18^, American Orthopedic Foot and Ankle Score (AOFAS)^19-22^: Used to determine whether rMPS alters analgesic perception after foot and ankle surgery. These instruments were applied on days 0 (before the first neuromodulation session), 5 (after the last neuromodulation session), 14, 30 and 90 after foot and ankle surgery.

### Data analysis

The following tests were used for statistical analysis of the collected data: Kruskal-Wallis test, Mann-Whitney test, Friedman test, Wilcoxon test; Spearman correlation; confidence interval for the mean and p-value.

### Ethics, funding, data sharing and disclosures

This study was approved by the Ethics Committee of the institution – protocol 73680223.3.0000.5142 and by the Brazilian Registry of Clinical Trials (REBEC) – Protocol RBR-8g8ckzm. All participants signed the Free and Informed Consent Form (FICF). The author guarantee that the data presented is accurate and transparent and that no important changes in study design or analyzes have been made after starting the analysis of the data. Data confidentiality was guaranteed in accordance with the General Data Protection Law (LGPD), Law No. 13,709/2018. There were no study sponsors or funding received for conducting the study.

## RESULTS

Of the participants, 61.2% were female (n=41) and 38.8% male (n=26), with a mean age of 51.7 years (±15.1). Most surgeries were bone interventions (74.6%, n=50); soft tissue procedures accounted for 25.4% (n=17). Forefoot surgery was the most prevalent (37.3%, n=25), followed by ankle (35.8%, n=24), hindfoot (19.4%, n=13), and midfoot (7.5%, n=5) (Table 1).

**Table 1:** Characterization of the general study sample.

		N	%
Group	Control	33	49.3%
	Experimental	34	50.7%
Gender	Female	41	61.2%
	Male	26	38.8%
Surgery	Bone	50	74.6%
	Soft tissue	17	25.4%
Area	Forefoot	25	37.3%
	Midfoot	5	7.5%
	Hindfoot	13	19.4%
	Ankle	24	35.8%
Smoking	No	57	85.1%
	Yes	10	14.9%
Functionality	Active	21	31.3%
	Sedentary	46	68.7%
Surgery time	1h	31	46.3%
	2h	31	46.3%
	3h	4	6.0%
	4h	1	1.5%
Anesthesia	Block and sedation	7	10.4%
	Spinal and sedation	60	89.6%
Tourniquet	No	7	10.4%
	Yes	60	89.6%

Legend: N: Total number.

Source: Study author.

Arthroscopic reconstruction for lateral ankle instability was the most common surgery performed (14.9%, n=10), followed by Chevron/Akin hallux valgus correction (13.4%, n=9) and neurectomy for Morton's neuroma (10.4%, n=7). In evaluating postoperative pain following foot and ankle surgeries, the average DN4 score was 3.40 (±1.54), ranging from 2 to 8. Applying the established threshold of 4 for identifying pain with a neuropathic component, the findings demonstrate that postoperative pain most frequently exhibited nociceptive characteristics. DN4 scores did not significantly differ between operated regions (forefoot, midfoot, hindfoot, ankle; p=0.794), indicating postoperative nociceptive pain was consistent across foot and ankle segments (Table 2).

**Table 2:** Comparison between the anatomical area of surgery and the DN4 score.

	Mean	Median	SD	N	IC	P-value
Forefoot	3.68	3	1.84	25	0.72	0.794
Midfoot	4.00	4	2.00	5	1.75	
Hindfoot	3.31	3	1.65	13	0.90	
Ankle	3.04	3	0.91	24	0.36	

Legend: CI: Confidence interval. N: Total number. SD: Standard Deviation.

Source: Study author.

**Figure 2:**
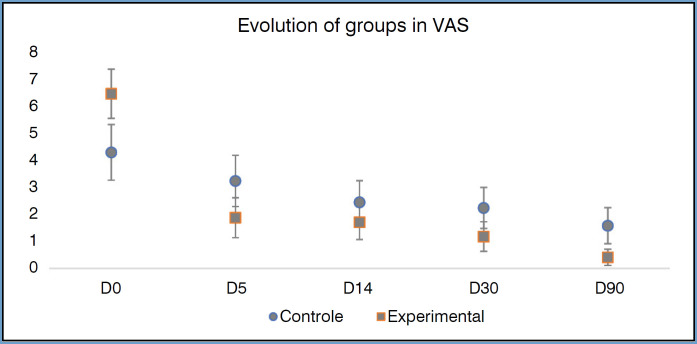
Evolution of groups in VAS.

Legend: D: Day.

Source: Study author.

Pain scores on the VAS dropped significantly faster in the EG compared to the CG. Although the EG started with higher pain (6.47 vs. 4.3), it showed a rapid decline from D5 onward, while the CG's reduction was slower. Statistically significant differences between the groups emerged from D5, as shown by the confidence intervals (Figure 2).

**Figure 3:**
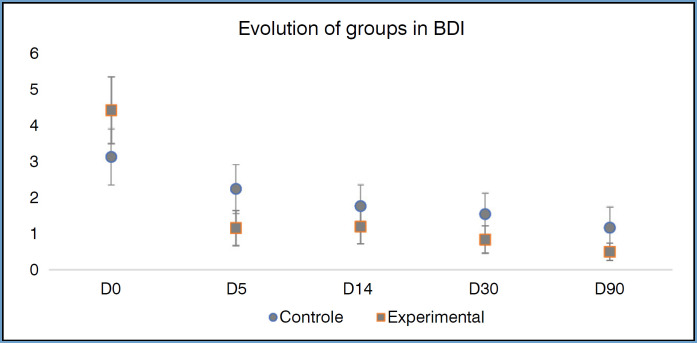
Evolution of groups in BDI.

Legend: D: Day.

Source: Study author.

The BDI results followed a similar pattern to the VAS. On D0, the EG presented a higher score (4.44) than the CG (3.14). From D5 onwards, there was a reduction in the EG, while the CG maintained a slower decrease. The CIs show that the differences between the groups are significant (Figure 3).

**Figure 4:**
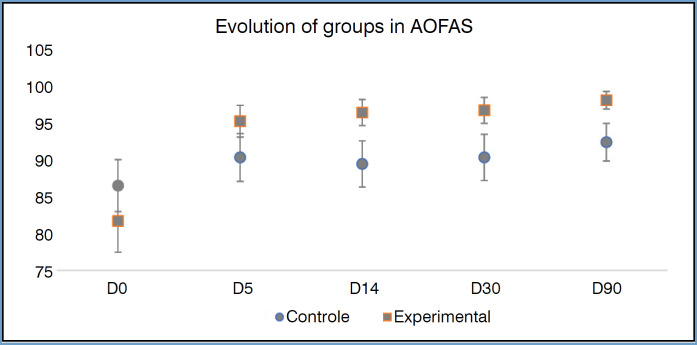
Evolution of groups in AOFAS.

Legend: D: Day.

Source: Study author.

Regarding the AOFAS, on D0, the CG presented a higher score (86.7) than the EG (81.8), indicating a possible initial difference. However, from D5 onwards, the EG surpassed the CG, reaching significantly higher scores compared to the CG. The CIs demonstrate that these differences are statistically relevant (Figure 4).

VAS, BDI and AOFAS scores between groups on postoperative days 0, 5, 14, 30, and 90 were compared (Table 3).

**Table 3:** Intergroup comparison of VAS, BDI and AOFAS scores on days 0, 5, 14, 30 and 90 postoperatively.

		Mean	Median	SD	Q1	Q3	IQR	N	IC	P-value
VAS 0	Control	4.30	5	3.03	2	6	4	33	1.03	0.005
	Experimental	6.47	6.5	2.72	5	8	3	34	0.91	
VAS 5	Control	3.24	3	2.77	1	5	4	33	0.95	0.034
	Experimental	1.88	1	2.20	0	3	3	34	0.74	
VAS 14	Control	2.45	2	2.35	0	5	5	33	0.80	0.225
	Experimental	1.71	1	1.92	0	3	3	34	0.64	
VAS 30	Control	2.24	2	2.22	0	5	5	33	0.76	0.049
	Experimental	1.18	0	1.64	0	2	2	34	0.55	
VAS 90	Control	1.58	0	1.97	0	3	3	33	0.67	0.006
	Experimental	0.41	0	0.89	0	0	0	34	0.30	
BDI 0	Control	3.14	3	2.28	1.36	4.27	2.91	33	0.78	0.035
	Experimental	4.44	5	2.76	2.79	7	4.21	34	0.93	
BDI 5	Control	2.25	2	1.99	0.9	3	2.1	33	0.68	0.013
	Experimental	1.16	0.36	1.45	0	2.07	2.07	34	0.49	
BDI 14	Control	1.77	1.63	1.72	0	3	3	33	0.59	0.138
	Experimental	1.20	0.95	1.43	0	2	2	34	0.48	
BDI 30	Control	1.55	1.18	1.71	0	2	2	33	0.58	0.135
	Experimental	0.84	0	1.12	0	1.27	1.27	33	0.38	
BDI 90	Control	1.17	0.63	1.67	0	1.63	1.63	33	0.57	0.129
	Experimental	0.50	0	0.71	0	1	1	34	0.24	
	Experimental	76.3	86	29.6	60.5	100	39.5	34	9.9	
AOFAS 0	Control	86.7	90	10.5	80	90	10	33	3.6	0.142
	Experimental	81.8	85	12.9	72.5	90	17.5	34	4.3	
AOFAS 5	Control	90.6	90	9.7	90	100	10	33	3.3	0.017
	Experimental	95.6	100	6.6	90	100	10	34	2.2	
AOFAS 14	Control	89.7	90	9.5	80	100	20	33	3.2	0.001
	Experimental	96.8	100	5.3	90	100	10	34	1.8	
AOFAS 30	Control	90.6	90	9.3	90	100	10	33	3.2	0.001
	Experimental	97.1	100	5.2	92.5	100	7.5	34	1.8	
AOFAS 90	Control	92.7	90	7.6	90	100	10	33	2.6	<0.001
	Experimental	98.5	100	3.6	100	100	0	34	1.2	

Legend: AOFAS: American Orthopaedic Foot and Ankle Society. VAS: Visual Analog Scale. BDI: Brief Pain Inventory. CI: Confidence Interval. CV: Coefficient of Variation. IQR: Interquartile Range. N: Total number. Q1: 1st Quartile. Q3: 3rd Quartile. SD: Standard Deviation.

Source: Study author.

The comparison of opioid consumption between the CG and EG constitutes one of the main outcomes of this study, objectively reflecting the effectiveness of electromagnetic neuromodulation in postoperative pain control. In the first postoperative week, mean opioid use was similar between groups (CG: 2.91 ± 2.38 tablets; EG: 3.62 ± 2.44; p=0.217). By the second week, opioid consumption remained notable in the CG (1.97 ± 2.16), while the EG had a significant reduction (0.50 ± 0.75; p=0.002). The IQR further highlights this, with just 1 tablet in the EG versus 3 in the CG, indicating more consistent lower opioid use among experimental group (Table 4).

**Table 4:** Comparison between groups for opioid consumption.

		Mean	Median	SD	Q1	Q3	IQR	N	IC	P-value
Opioid 1st week	Control	2.91	2	2.38	1	4	3	33	0.81	0.217
	Experimental	3.62	3	2.44	2	5.75	3.75	34	0.82	
Opioid 2nd week	Control	1.97	2	2.16	0	3	3	33	0.74	0.002
	Experimental	0.50	0	0.75	0	1	1	34	0.25	

Legend: CI: Confidence Interval. IQR: Interquartile Range. N: Total Number. Q1: 1st Quartile. Q3: 3rd Quartile. SD: Standard Deviation.

Source: Study author.

## DISCUSSION

Analysis of the total sample of study participants (n=67) showed that, based on the DN4 score, postoperative pain in foot and ankle surgeries was primarily nociceptive (3.40; ±1.54), regardless of the affected region (ankle, hindfoot, midfoot, or forefoot). This finding highlights the importance of tailoring treatment to control acute pain and suggests a relevant inflammatory and mechanical profile for analgesic choice. Orthopedic surgeries generally result in more postoperative pain than other types of operations^23^. Chung, Ritchie and Su^24^ studied 10,008 patients and found orthopedic procedures led to the highest rates of reported pain. Factors such as body mass index, length of anesthesia, and specific surgery types can predict severe pain, though this study did not find anesthesia type to be influential. Similarly, McGrath et al.^25^ examined 5,703 patients and noted that outpatient orthopedic procedures ranked among the most painful, with ankle surgeries accounting for two of the seven worst cases.

The clinical trial assessed whether rMPS impacts postoperative pain in foot and ankle surgeries. VAS scores dropped from 5.40 (Day 0) to 0.99 (Day 90), showing significant improvement. BPI scores also decreased from 3.80 to 0.83 over the same period, but variability among patients remained high. AOFAS scores rose from 84.2 (Day 0) to 95.7 (Day 90), also indicating better pain control.

rEMP has proven to be a useful tool that can be used in postoperative pain control in foot and ankle surgeries^10,12,26^. Regarding electromagnetic neuromodulation applied in foot and ankle surgeries, a study by Gerdesmeyer et al.^27^ evaluated electromagnetic neuromodulation in 53 patients with chronic Achilles tendinopathy, comparing a treated group (80 mT, 3 Hz, 8 sessions) and another using elevation insoles. Both groups showed significant pain reduction, with a greater reduction in the group undergoing neuromodulation (VAS from 6.9 ± 1.3 to 3.6 ± 2.0; p < 0.01), especially from the 5th day onward. Despite focusing on a chronic condition, the study highlights the effectiveness of neuromodulation in foot and ankle pathologies, reinforcing its potential, even with differences in the clinical contexts analyzed.

Saltzman et al.^28^ evaluated peripheral electromagnetic neuromodulation as an adjunctive treatment for delayed union in 19 foot and ankle arthrodeses. Using a protocol with immobilization, limited weight-bearing, and electromagnetic stimulation, they observed low effectiveness of the technique alone, with only 26% success rate (5/19 cases). Although the objective of the current study was different, analyzing postoperative pain, this is one of the few studies in the foot and ankle field.

Tassone et al.^29^ conducted a randomized, double-blind trial of peripheral electromagnetic neuromodulation in 182 diabetic neuropathy patients, dividing them into experimental (n=92) and control (n=90) groups. The experimental group had a 30% pain reduction, with 48% responding to treatment vs. 28% in control (p<0.05), though opioid use did not differ significantly between groups (p>0.05). Neuromodulation led to quicker and greater pain relief; differences in drug response were attributed to varying conditions analyzed. While the study by Tassone et al. confirmed neuromodulation's effectiveness for chronic peripheral neuropathy, the results of this clinical trial showed its value as an adjuvant in multimodal postoperative analgesia for foot and ankle surgeries involving acute pain. Neuromodulation has thus demonstrated analgesic potential in multimodal analgesia.

Another relevant point to this study concerned the use of opioids. In the total sample (n=67), mean opioid consumption was similar between the CG (2.91±2.38) and EG (3.62±2.44) in the first postoperative week (p=0.217). In the second week, there was a significant reduction in the EG (0.50±0.75) compared to the CG (1.97±2.16), with p=0.002. Electromagnetic neuromodulation contributed to reduced postoperative opioid use, highlighting its role in multimodal analgesia. From a clinical perspective, the reduced need for opioids corroborates the benefit of neuromodulation, the subject of this study. Opioids are considered an alternative treatment and potentially harmful to health, especially when used for long periods. In this way, EMPr contributes to postoperative safety, in addition to aligning with current guidelines for rationalizing the use of opioids^30^.

Among the study's limitations are: the scarcity of specific literature investigating the combination of peripheral electromagnetic neuromodulation with postoperative pain control in patients undergoing foot and ankle surgery; the grouping of different types of foot and ankle surgeries into a single group, without differentiating between major and minor procedures, bone versus soft tissue surgeries, or more complex versus simpler interventions; the heterogeneity of peripheral electromagnetic neuromodulation protocols used in the literature represents a significant limitation for the comparative analysis and discussion of the results.

## CONCLUSION

Peripheral electromagnetic neuromodulation applied in the postoperative period of foot and ankle surgeries demonstrated efficacy in reducing pain and opioid consumption compared with the control group. The three assessment scores used — VAS, IBD, and AOFAS — showed consistent improvement throughout follow-up, indicating progressive pain relief. The DN4 questionnaire analysis showed that the predominant pain pattern was nociceptive, reinforcing the role of neuromodulation in peripheral modulation of postoperative pain. These findings support the use of this technique as a safe and effective adjuvant strategy for pain management in orthopedic foot and ankle surgeries.

## Data Availability

The underlying contents of the research text are contained in the manuscript.
